# AI-Assisted High-Throughput Tissue Microarray Workflow

**DOI:** 10.3390/mps7060096

**Published:** 2024-11-25

**Authors:** Konrad Kurowski, Sylvia Timme, Melanie Christine Föll, Clara Backhaus, Philipp Anton Holzner, Bertram Bengsch, Oliver Schilling, Martin Werner, Peter Bronsert

**Affiliations:** 1 Institute for Surgical Pathology, Medical Center, Faculty of Medicine, University of Freiburg, 79106 Freiburg, Germany; 2 Core Facility Histopathology and Digital Pathology Freiburg, Medical Center, University of Freiburg, 79106 Freiburg, Germany; 3 Tumorbank Comprehensive Cancer Center Freiburg, Medical Center, University of Freiburg, 79106 Freiburg, Germany; 4 Department of Obstetrics & Gynecology Medical Center, University of Freiburg, 79106 Freiburg, Germany; 5 Department of General and Visceral Surgery, Medical Center, Faculty of Medicine, University of Freiburg, 79106 Freiburg, Germany; 6 Clinic for Internal Medicine II, Gastroenterology, Hepatology, Endocrinology, and Infectious Disease, Medical Center, University of Freiburg, 79106 Freiburg, Germany

**Keywords:** immunohistochemistry, tissue microarrays, AI, workflow, FFPE tissue

## Abstract

Immunohistochemical (IHC) studies of formalin-fixed paraffin-embedded (FFPE) samples are a gold standard in oncology for tumor characterization, and the identification of prognostic and predictive markers. However, despite the abundance of archived FFPE samples, their research use is limited due to the labor-intensive nature of IHC on large cohorts. This study aimed to create a high-throughput workflow using modern technologies to facilitate IHC biomarker studies on large patient groups. Semiautomatic constructed tissue microarrays (TMAs) were created for two tumor patient cohorts and IHC stained for seven antibodies (ABs). AB expression in the tumor and surrounding stroma was quantified using the AI-supported image analysis software QuPath. The data were correlated with clinicopathological information using an R-script, all results were automatically compiled into formatted reports. By minimizing labor time to 7.7%—compared to whole-slide studies—the established workflow significantly reduced human and material resource consumption. It successfully correlated AB expression with overall patient survival and additional clinicopathological data, providing publication-ready figures and tables. The AI-assisted high-throughput TMA workflow, validated on two patient cohorts, streamlines modern histopathological research by offering cost and time efficiency compared to traditional whole-slide studies. It maintains research quality and preserves patient tissue while significantly reducing material and human resources, making it ideal for high-throughput research centers and collaborations.

## 1. Introduction

Tissue microarrays (TMAs) consist of multiple tissue cores derived from formalin-fixed paraffin-embedded (FFPE) tissue samples on a single slide. TMAs enable high-throughput analysis of multiple samples under standardized conditions with high reproducibility. This innovative approach, first described by Wan et al. in 1987 [1], enables the aggregation of multiple tissue samples in a single paraffin block, the TMA block. Cores measuring between 0.6 and 2.0 mm are extracted from FFPE tissue blocks and arranged within a new paraffin block in a grid pattern. Compared to conventional whole-slide tissue sections, TMAs are more economical and efficient, saving material, reagents, antibodies, human resources and preserving tissue samples. In the processing of tissues for biochemical methods such as immunohistochemistry (IHC) and mass spectrometry imaging [2,3], TMAs provide a more consistent evaluation due to reduced variability [4,5,6].

Biobanks play a crucial role in biomedical research involving patient tissues and have emerged as a viable option for sourcing patient samples for the creation of TMAs. They are instrumental in the collection, storage, and processing of patient samples, providing technological platforms for research collaborations. These collaborations extend to project planning and execution, positioning biobanks as intermediaries between pathologists, clinicians, researchers, and patient samples. In research collaborations, patient tissues are procured from archives and processed according to the project design. Moreover, biobanks provide sample-specific clinicopathological data such as WHO- and UICC-classification and patient follow-up in a pseudonymized manner.

Despite biobanks being accessible for research collaborations, a considerable amount of scientific potential remains untapped in archived tissue samples. Limiting factors include time-consuming procedures, and high personnel needs for IHC studies, especially when human resources are limited. The medical field faces a shortage of pathologists amidst rising workloads due to an aging population and increasing diagnostic complexity [7].

Digital pathology, supported by AI, offers a promising solution [8,9]. Advances in whole-slide scanning technology have enabled the digitization of microscopic tissue examination and the subsequent inclusion of AI. Studies show that AI-assisted pathologists achieve higher diagnostic accuracy than either AI or pathologists alone [10,11,12,13,14]. AI algorithms are also implemented in research to help interpret tissue samples, offering improved accuracy and reduced inter-observer variability [15]. AI is yet to be broadly applied in routine diagnostics, but promising or even certified solutions are already available [8,16,17,18], suggesting that AI’s role in pathology is set to grow.

However, a reliable workflow integrating these techniques for large-scale patient studies is not yet established. The primary goal of the ensuing study was to develop and optimize a resource-efficient workflow using advanced hardware and software to conserve resources while simultaneously meeting state-of-the-art scientific standards.

## 2. Materials and Methods

### 2.1. Patient Tissue

Two patient cohorts (Cohort A and Cohort B) were included in the presented study. All FFPE tumor samples were derived from the archives of the Institute for Surgical Pathology (ISP) and the Biobank of the Comprehensive Cancer Center Freiburg (CCCF Biobank), both located at the University Medical Center Freiburg, Germany. All patients included in the study underwent surgery at the University Medical Center Freiburg, Germany. Informed consent was obtained from all patients prior to inclusion. Both presented studies were approved by the local Ethics Committee of the University of Freiburg (REF 21-1684 and REF 20-289). All tissue samples were processed and diagnosed at ISP according to guideline-conformant and standardized gross sectioning protocols. Cohort A consisted of 49 patients with intrahepatic cholangiocellular carcinoma. Cohort B consisted of 142 patients with non-small cell lung cancer, which were subdivided into subcohorts of 78 patients with adenocarcinoma (B1) and 64 patients with squamous cell carcinoma (B2). All cases were confirmed during primary diagnosis using IHC staining (Cytokeratin 7, Cytokeratin 5/6, p40, thyroid transcription factor 1).

Tumor-containing FFPE tissue specimens were sectioned (using the Leica RM 2255 microtome, Leica Biosystems, Nussloch, Germany), stained with hematoxylin–eosin (H&E) (using the TissueTek Prisma autostainer, Sakura Finetek Europe, Alphen aan den Rijn, The Netherlands), and histologically reviewed by board-certified pathologists before inclusion.

Clinicopathological data were automatically extracted from the ISP’s structured reports (SRs) of oncological patient samples, established by Aumann et al. [19,20,21]. In short, these template-based SRs are organized into distinct text blocks containing WHO- and UICC-classification relevant data, including histological subtype, pT-, pN-, pM-, L-, V-, Pn-status, tumor size, number of lymph node metastases, distant metastases, and surgical margin status. To generate the histopathological reports, all relevant data were entered into predefined templates, leading to the automatic creation of the pathological reports. Simultaneously, an integrated electronic form linked to the cancer registry database was compiled in accordance with the Early Detection of Cancer and Cancer Registries Act [22] introduced in 2015 in Germany, which mandates the systemic documentation of malignancies.

Patient outcome was obtained by request through the University Medical Center Freiburg’s computational center. Data were compiled and securely stored on a user-restricted network drive. Next, every tissue specimen was pseudonymized with a unique identifier. To ensure an unequivocal sample-to-patient linking, a barcode-based pseudonymization was developed. Therefore, FFPE blocks and corresponding slides were labeled with barcodes generated using the BarCode Generator software (Bytescouts, v. 7.3). The labels comprised Data Matrix barcodes ciphering 6–9 characters. Adjacent to the barcodes, the caption was visualized in Arial font, size 15.75. All labels were printed using a Zebra ZD421 printer (Zebra Technologies Corporation, Lincolnshire, IL, USA) on precision-fit labels of 23 × 8 mm.

### 2.2. Annotation of Regions of Interest

All H&E-stained slides were digitized using a Pannoramic 1000 scanner (3DHISTECH Ltd., Budapest, Hungary) with a 20-fold microscope objective (20×, NA 0.8, Plan-Apochromat, 0.25 μm/pixel). After digitization, all images were reviewed for sufficient quality (focus, completeness, and artifacts). For the accurate transfer of specific tissue areas during TMA creation, punching areas were annotated using the CaseViewer software (Sysmex, v. 2.3). For both patient cohorts, circular regions of interest (ROI) of 1.0 mm in diameter were annotated within the tumor and the adjacent non-neoplastic tissue. Annotations were color-coded (tumor ROIs—red and non-neoplastic tissue—green) and labeled for distinction. If possible, up to three ROIs per patient per tissue type were annotated, although two tissue cores per tissue sample are considered representative, as shown in previous studies [23,24].

### 2.3. TMA Construction

**Figure 1:**
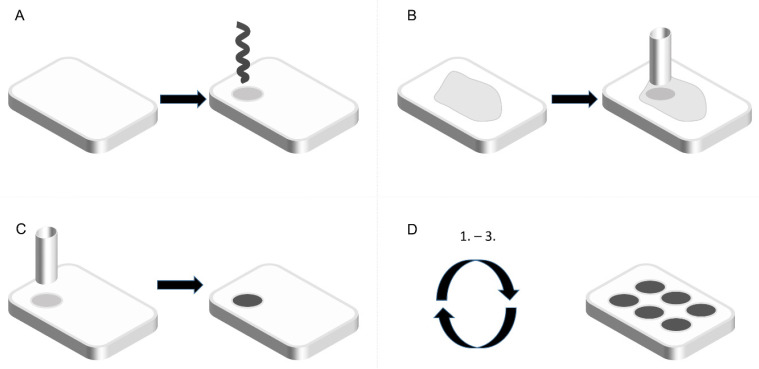
Schematic depiction of the TMA block construction. (**A**): A cylindrical cavity is drilled in the Acceptor block. (**B**): A tissue core is removed from the Donor block. (**C**): The core is embedded into the Acceptor block. (**D**): Repeated application results in the construction of a TMA. Source: Own illustration (PowerPoint, 2016).

Three repeatable steps define TMA construction: drilling of core holes into an empty paraffin block (Acceptor blocks) (Figure 1A), extraction of cores from FFPE blocks (Donor blocks) (Figure 1B), and their transfer into the Acceptor block (Figure 1C). This process is repeated, resulting in the construction of a TMA (Figure 1D).

### 2.4. Semi-Automated TMA Construction

**Figure 2:**
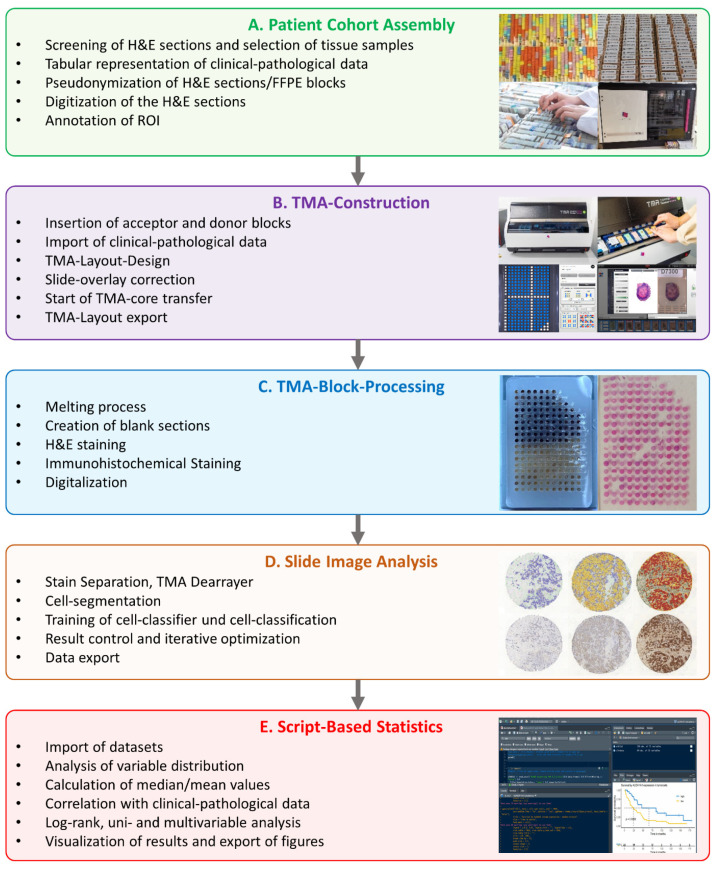
Schematic representation of the workflow for TMA construction with the TMA Grandmaster, AI-supported evaluation of antibody expression with QuPath, and script-based statistical analysis with RStudio. Source: Own illustration (PowerPoint 2016).

The TMA Grandmaster (TMA GM) is a semi-automated microarrayer that streamlines the construction of TMAs (Figure 2B). Upon setting up a new project in the TMA Control software, TMA GM’s block holders are loaded with Acceptor and Donor blocks. An integrated camera then captures images of these blocks, creating digital copies and identifying their barcoded pseudonyms for accurate tracking.

The selected TMA grid involved cores of 1.0 mm in diameter spread over a paraffin block of 24 × 37 mm with a minimal spacing of 0.4 mm. By selectively deactivating certain spots in the layout, an asymmetric grid was set. The transfer process was set up in a random sequence within specific TMA blocks. The arrangement of patients across the TMA blocks was sequential. Next, digital slides, including the previously annotated ROIs, were automatically overlaid onto the digital Donor blocks and manually aligned. The punch and transfer processes were initiated once the ROIs were confirmed for transfer into the Acceptor block. After the transfer, TMA data encompassing core localization and clinical information were exported for precise sample identification. As a part of external quality controls, tonsil, and placenta tissue samples with verified antibody expression were included in the TMA layout to ensure the sensitivity and specificity of subsequent analyses (e.g., IHC stains). Each cohort was allocated to separate TMA blocks. For Cohort A, two TMA blocks were created. Cohort B encompassed six TMA blocks. Additionally, a second set of TMAs, referred to as TMA Clones, was created for each study. These clones had an identical layout to the original TMAs but included spatially adjacent tissue cores. This was achieved by creating a second set of annotations next to the primary ROIs and subsequently transferring these cores into the TMA Clones.

### 2.5. TMA Block Processing

After the punching process, the TMA blocks were melted to prevent tissue loss during the subsequent sectioning and IHC staining (Figure 2C).

For melting the TMA blocks, protocols already described in the literature [25,26,27,28] were tested, refined, and optimized. The adapted melting process involved a series of multiple incubation cycles at 37 °C for 60 min, interspersed with active cooling of the samples on ice for ten minutes. This step was performed three times for Cohort A and six times for Cohort B.

### 2.6. Immunohistochemistry

Tissue sections of 2 µm thickness (Leica RM2255 rotary microtome, Leica Biosystems, Nussloch, Germany) were prepared from the melted TMA blocks and deparaffinized. One section per TMA was stained with H&E. Further sections were IHC stained using the EnVision FLEX detection system (Dako, K8000, Agilent Technologies, Santa Clara, CA, USA) and commercially purchased primary antibodies.

From Cohort A, two of the three TMAs were IHC stained with the following six antibodies each: Anti-Biglycan (BGN, Abcam, monoclonal rabbit antibody, (EPR20235)), Anti-Histone H1.0 (H1, Abcam, monoclonal rabbit antibody, (EPR6537)), Anti-Kruppel-like factor 7 (KLF7, Sigma Aldrich, polyclonal rabbit (HPA030490)), Anti-Lactate Dehydrogenase antibody (LDH1A1, Abcam, monoclonal rabbit antibody (EP1566Y)), Anti-SKI (SKI, Invitrogen, polyclonal rabbit antibody (PA5-51253)), Anti-Vitronectin/S-Protein antibody (VN, Abcam, monoclonal rabbit Antibody (EP873Y)).

From Cohort B, all six TMAs were stained with anti-ubiquitin-like modifier-activating enzyme 1 (UBA1, Invitrogen, Recombinant Rabbit Monoclonal Antibody (SD08-62)). The staining protocol included the following steps: antigen unmasking with retrieval solution (UBA1, SKI: sico 5, five minutes; BGN, H1, KLF7, VN: sico 2, two minutes; LDH1A1: pH 6.1, 30 min; ten-minute incubation with blocking reagent (EnVision FLEX Peroxidase Blocking Reagent, DAKO), staining with the above-mentioned primary antibodies at a respective concentration of 4.0 µg/mL (UBA1), 0.5 µg/mL (BGN, H1, SKI, VN), 1:100 (KLF7, concentration not specified), 1:1000 (LDH1A1, concentration not specified) and 60 min. incubation at room temperature each, 15 min. incubation with Mouse Linker (EnVision Flex + Mouse Linker, DAKO), 20 min. incubation with peroxidase (EnVision FLEX/HRP, DAKO), 10 min. incubation with Envision FLEX DAB + Substrate Buffer (1:51) and finally counterstaining with hematoxylin. Subsequently, all stains were verified for specific antibody expression, using both internal and external controls, and digitized as previously described.

### 2.7. QuPath Analysis

The analysis of the IHC staining was performed using QuPath (v0.3.0) [29] (Figure 2D). Initially, stain separation was performed to separate the color spectrum into individual color channels and to enable accurate quantitative analysis. Individual cores were detected automatically with the “TMA-Dearrayer” function and overlaid with a digital grid. The TMA layout was imported for sample-pseudonym annotation of every single core.

To quantify antibody expression in tissue marked by DAB staining, individual cells were detected using QuPath’s ‘Positive cell detection’. Key settings included ‘Detection image’ to guide cell nucleus identification, with options like ‘Hematoxylin OD’ for cytoplasmic AB staining and ‘Optical density sum’ for nuclear AB staining. ‘Minimum and maximum area’ settings defined nuclear size limits and were also used for excluding artifacts. ‘Threshold’ identified nuclei based on color intensity, adjusted for faint staining. ‘Max background intensity’ excluded high-intensity non-nuclear elements. ‘Cell expansion’ defined the extension of the simulated cell membrane around nuclei. ‘Intensity threshold parameters’ and ‘Score compartment’ were crucial for detecting DAB staining, varying based on nuclear or cytoplasmic expression patterns. ‘Single Threshold’ categorized cells as DAB-positive or -negative, with adjustable intensity levels (1+, 2+, 3+). Settings were tested on various TMA cores, visually inspected for segmentation and DAB-detection accuracy, and adjusted iteratively in QuPath to optimize cell segmentation. Ultimately, the trained model was applied to all TMA cores. To distinguish cell types, an ‘Object Classifier’ was trained by manually annotating cell groups to the tissue classes “Tumor” and “Stroma”. QuPath extracted features from these annotated cells for further classification, including morphological characteristics like size, shape, or texture. For Cohort A, 1088 annotations with a cumulative area of 5.11 mm^2^ covering 42,308 cells were assigned; for Cohort B, 501 annotations covering 19.63 mm^2^ with 144,927 cells were assigned. These manually classified cells served as training data for the classification model using the Random Tree machine learning algorithm. The model was trained with extracted features to understand patterns and relationships between features and target classes, assigning each cell to appropriate classes. Results were visually inspected and refined iteratively by manually annotating wrongly classified tissue areas, further enhancing the classifier before it was applied across all TMA cores.

Finally, all metrics for each core were exported as a .tsv file, including the absolute expression percentage within the tumor and surrounding stromal cells. Additionally, the H-Score, quantifying both intensity and percentage of antibody expression, was automatically calculated and displayed on a scale of 0–300.

### 2.8. Statistical Analysis with RStudio

All statistical analyses were automated through a custom script coded with R (v4.0.3; R Core Team 2020) in the graphical interface RStudio (v2023.03.0+386 RStudio Team (2020). RStudio: Integrated Development for R. RStudio, PBC, Boston, MA, USA), which compiled the QuPath results of antibody expression with clinicopathological data (Figure 2E). Interested readers can be granted access to the code upon request. The script performed the following tasks:

1. Importing both clinicopathological data and QuPath analyses into R (readxls).

2. Sorting QuPath results by patient and calculating variable distribution as well as mean and median values per patient and tissue type (dplyr).

3. Compilation of these values with clinicopathological data into a new dataset (R Base Package).

4. Calculation of an optimal cutoff for antibody expression and dichotomizing into high and low expression subgroups (cutpointr).

5. Generation of survival curves and statistical significance assessment via log-rank testing (survival).

6. Visualization of Kaplan–Meier curves (survminer).

7. Univariable and multivariable Cox regression analyses for hazard ratios (survival; gtsummary).

8. Tabular visualization of the Cox regression (gtsummary).

9. Export of graphs, tables, and Kaplan–Meier curves in various formats (JPEG, PNG, PDF, or DOCX).

## 3. Results

### 3.1. Pseudonymization with Barcoded Labels

The barcode reading feature of the TMA GM utilizes a built-in camera rather than an infrared scanner, resulting in reduced barcode legibility in conditions of poor contrast, low quality, or contamination, such as paraffin deposits. This made it inadequate for reading imprinted barcodes on paraffin cassettes with a detection rate of <10% under present conditions. The development of DataMatrix barcode labeling for Donor blocks significantly increased detection rates to at least 83% (two-sample *t*-test, two-tailed: *p* < 0.001; Table 1). This ensured a reliable association of each core to its respective Donor block as well as the simultaneous pseudonymization of the cohort.

A Zebra ZD421 printer was used to create custom, non-glossy, non-permanent labels of 23 × 8 mm in size. These labels did not interfere with the existing identifying codes on the tissue blocks and ensured easy, residue-free removal.

In essence, switching to DataMatrix barcoded labels improved detection rates to over 83%, ensured reliable core-to-Donor-block association, and successfully pseudonymized patient samples.

### 3.2. TMA Layout

**Figure 3:**
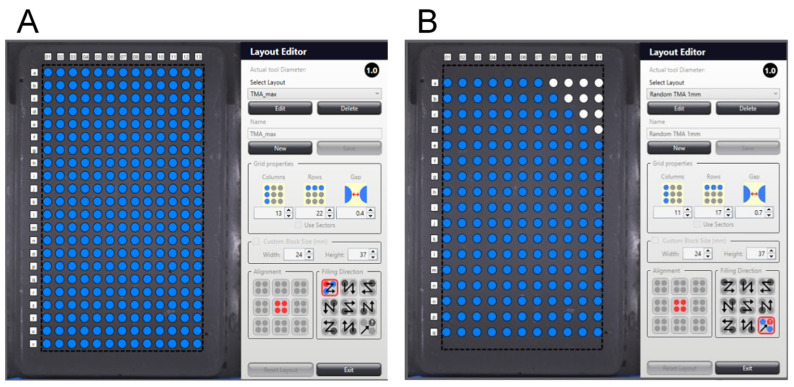
Examples of TMA layouts. (**A**) A suboptimal symmetrical TMA layout with linear core arrangement and minimal spacing (0.4 mm) between cores. This layout accommodates the maximum number of cores (n = 286 with 1 mm core diameter) but complicates spatial orientation and may lead to core overlaps during microtome cutting. (**B**) An asymmetrical TMA layout with random core arrangement. The upper right corner is deactivated for punching and aids in orientation. The increased spacing of 0.7 mm between cores prevents overlaps during cutting but reduces the maximum number of cores (n = 177). Source: Own illustration.

The TMA layout was meticulously designed based on the required number of core samples, as demonstrated in Figure 3. If the anticipated amount of core samples was below the TMA’s full capacity, the layout editor was initially adjusted by decreasing the rows and columns (thus limiting the potential core count), allowing increased spacing between cores. Minimum spacing (0.4 mm) frequently caused cores to overlap upon dislodging during microtome cutting. Overlapping, in turn, resulted in certain cores becoming unfit for evaluation, thus reducing the sample size. To prevent overlapping, distances of 0.7–0.8 mm between cores were chosen. Moreover, an asymmetrical layout was implemented for precise spatial orientation and accurate macroscopic allocation of punch samples; this was accomplished by deactivating core transfer in spots in the upper right corner of the layout. Random core arrangement in the layout was chosen to normalize batch effects.

Substantially, the TMA layout was meticulously designed to optimize core spacing and prevent overlap, ensuring precise sample allocation and reducing evaluation issues.

### 3.3. Overlay of Digital H&Es and Digital Donor Blocks

**Figure 4:**
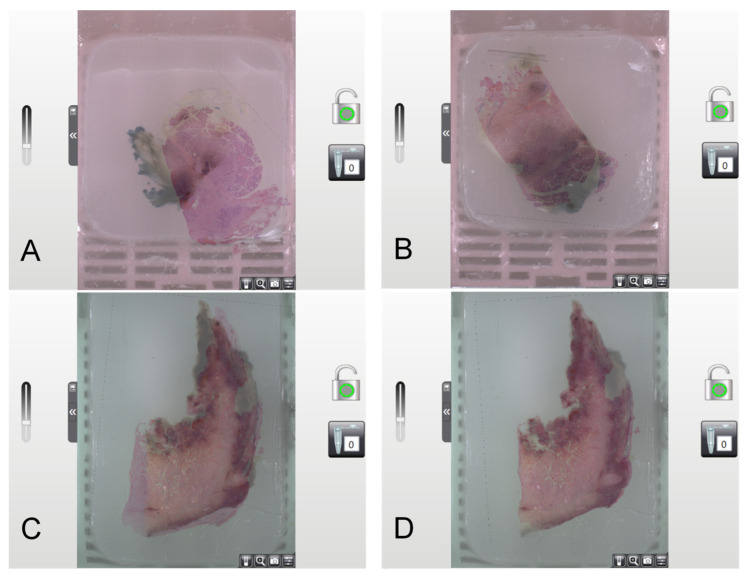
Examples of automatic overlay of H&E section image and digital Donor block: (**A**) Despite asymmetric and high-contrast tissue samples, the automatic overlay is incorrect. (**B**) Symmetrical, rectangular tissue samples often resulted in mirrored overlays. (**C**) Asymmetric and high-contrast samples led to more accurate automatic overlays; however, (**D**) minor adjustments remained necessary for exact alignment. Source: Own illustration.

The precise overlay of digital H&Es and the donor block is critical to TMA creation. Misalignment could lead to the extraction of a core that did not match the annotated position within the Donor block. Consequently, the tissue cylinder embedded in the TMA block would not correspond to the originally annotated ROI on the digital H&E image. Automated overlays by TMA Control Software (version 3.3) were inadequate, especially with low-contrast tissues like adipose material or symmetric samples, often resulting in misalignment (Figure 4A,B). High-contrast, asymmetric samples yielded better overlay accuracy (Figure 4C). Manual correction was required for all samples (Figure 4D). This represented a significant additional factor in terms of labor and time during the creation of the TMAs.

Essentially, accurate digital overlay between H&E images and Donor blocks is crucial for TMA creation, requiring manual correction due to software limitations, especially with low-contrast tissues.

### 3.4. Melting Process and Core Loss

Core loss refers to dislodgement or shift on the TMA slide during the slicing and/or staining process. Core loss occurs due to inadequate tissues-paraffin adhesion to the surrounding paraffin while being transferred from the Donor Block into pre-drilled cylindrical cavities in the Acceptor block, resulting in a visible gap. To re-establish adhesion, TMA blocks must undergo a melting process. Therefore, a protocol of alternating incubation at 37 °C and rapid cooling on ice was established. For Cohort A, a loss of 17 of 429 cores (4% loss) was observed in the H&E slides. For Cohort B, doubling the melting cycles reduced the loss rate significantly to an average of 1.25% (two-sample *t*-test, two-tailed: *p* = 0.001; Table 2).

Therefore, a method for significantly reducing core loss in TMA slides was successfully established through alternating incubation and cooling cycles.

### 3.5. Sectioning, Staining, and Digitization of TMAs

Sectioning TMAs with a microtome, IHC staining, and digitization were implemented as described above. The beneficial economic effects (in time and materials) compared to whole-slide studies are demonstrated in Table 3. In detail, for both cohorts, sectioning 18 slides took 60 min. Subsequent IHC staining required 720 min for both cohorts, including incubation times (456 min). Digitization of all TMAs took 135 min and 26.1 GB of storage space. In total, 5.4 µg of antibody were required (excluding LDH1A1 and VN due to lack of concentration information). For whole-slide consumables, the required time and disk space were significantly higher.

### 3.6. Staining Evaluation with QuPath

**Figure 5:**
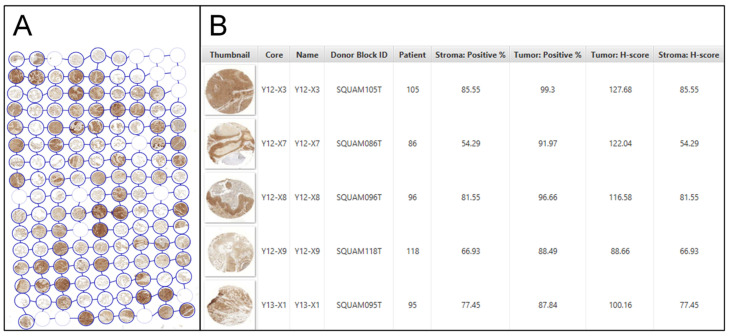
In QuPath, the TMA layout is adopted. Each core is annotated with the block and patient pseudonym. The location of the cores in the layout is indicated by X and Y coordinates (**A**). The antibody expression is presented both as a percentage and as an H-Score, with separate values provided for both tumor and stroma (**B**). Source: Own illustration.

The exported layout data ensured distinct mapping of each core to its respective patient (Figure 5A), annotated with the Donor block, and patient pseudonym. Antibody expression is reported as both a percentage and an H-Score, with separate values for tumor and stroma (Figure 5B). Cell segmentation settings yielded consistent results within the same cohorts and stains. The cell classifier for differentiating cell types was not universally applicable and needed retraining for each single TMA slide. On average, evaluating a stained TMA slice took 52.5 min, including manual verification of each core’s proper classification.

### 3.7. Correlation of Biomarker Expression and Clinicopathological Data with R

**Figure 6:**
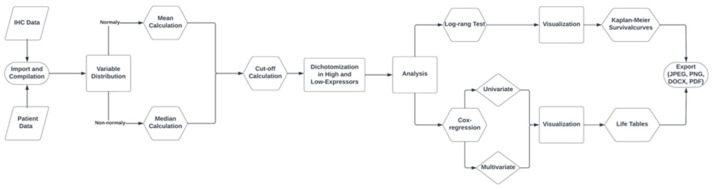
Workflow of custom coded R-script for statistical survival analysis of patient cohorts. Source: Own illustration (Lucidchart, v1.27.0).

**Figure 7:**
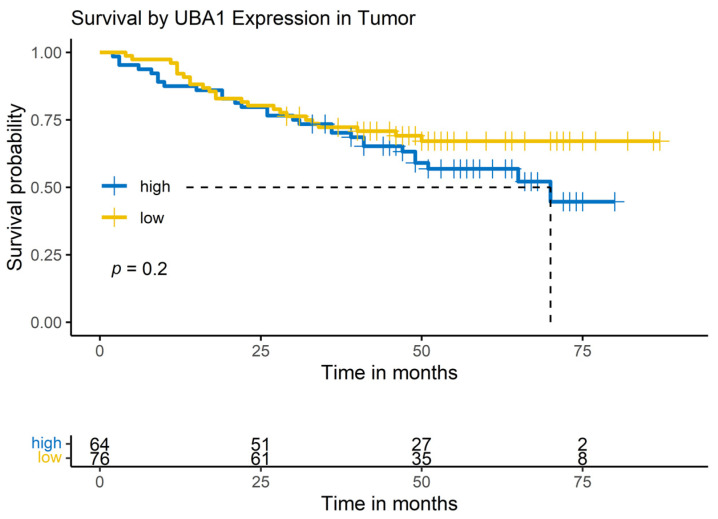
Example of result visualization. Log-rank models are exported as ready-to-use Kaplan–Meier curves. Source: Own illustration.

**Figure 8:**
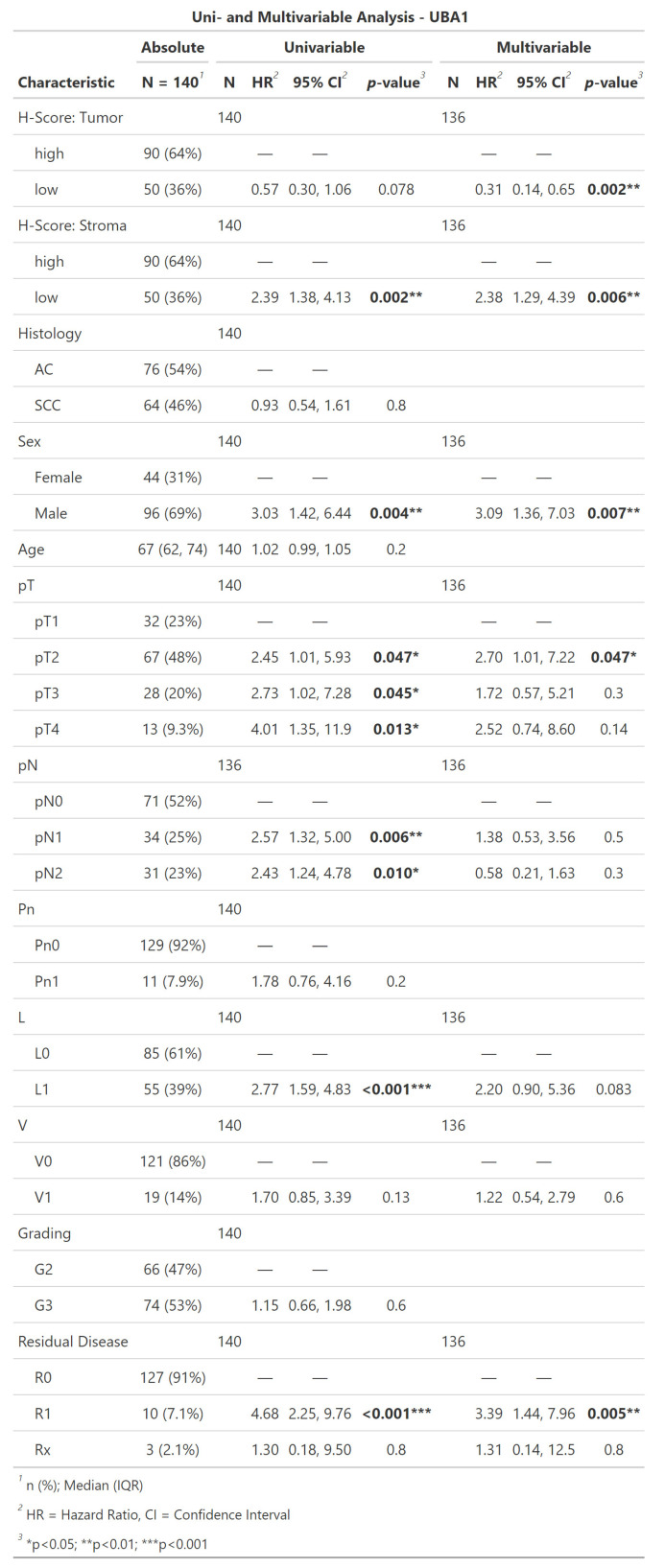
Example of result visualization. Uni- and multivariable Cox regression is reported in tables. Significant values are highlighted. Source: Own illustration.

All statistical calculations and visualizations were performed with the custom-coded script in R (Figure 6). The cohorts were dichotomized by an automatically calculated cut-off into high- and low-expressors. The impact of the biomarker expression on overall survival was analyzed per log-rank test. In uni- and multivariable Cox regression models, the biomarker expression was further analyzed for its effect on the hazard ratio. The yielding results were visualized by Kaplan–Meier curves and life tables in .png or .jpg files, as shown exemplarily in Figure 7 and Figure 8.

The workflow involved creating TMAs with a semi-automated tissue microarrayer from archived FFPE tissue samples, followed by AI-assisted analysis of the IHC-stained TMAs. The generated data is script-based correlated with clinicopathological data retrieved from the LIS and statistically analyzed. The results are graphically presented as ready-to-use figures and tables. The focus is the generation of valid, reproducible results while significantly reducing personnel and material resources compared to manual whole-slide studies. To accomplish this, a continuous workflow was established by aligning individual technology platforms in tissue processing, slide evaluation, and data analysis (Figure 2). This refined workflow was then applied to two patient-cohorts of different tumor types for proof of concept, assessing a total of seven potential biomarkers and statistically correlating them with corresponding clinicopathological data.

## 4. Discussion

The presented study introduces a scalable and replicable workflow for efficient IHC biomarker studies on large patient cohorts, beginning from FFPE tissue blocks to publication-ready results. The method optimizes the use of resources and is an alternative to traditional whole-slide IHC studies. The workflow allows for quicker result generation—saving time and cost—and increasing productivity and efficiency while maintaining result reliability.

For the proof of concept, it was applied and validated on two tumor patient cohorts. The workflow relies on three components:

a. Semi-automatically created TMAs, available through existing patient consent forms and a positive ethical vote for research projects, annotated with clinicopathological data.
b. AI-supported evaluation of AB expression of these TMAs.
c. Script-based correlation of AB expression with clinicopathological parameters and statistical analysis, resulting in immediate generation of publication-ready figures and tables.

Compared to a whole-slide study, the presented workflow significantly reduced material and labor resources. Labor time decreased to 7.7% (34.00 vs. 438.11 h). Only 18 unstained slides (2% of the whole-slide study’s 851) were required, taking 15.25 h to process versus 347.49 h for whole-slide studies (4.3%). Antibody use was also significantly lower (5.4 µg vs. 271.3 µg, or 1.9%). Labor for sample evaluation and analysis was cut to 20.6% (18.75 vs. 90.6 h). “Material loss” of patient tissue was minimized, preserving FFPE samples for potential future diagnostic investigations while reducing the number of slides improved digitization speed, storage needs, and computational analysis efficiency.

The resulting resource savings correlate with high initial costs. Despite the undeniable expenses associated with acquiring equipment such as the TMA Grandmaster, barcode printers for slides and capsules, slide scanners, and PCs, digital pathology is advancing steadily. This progress is reflected in innovations like barcode-driven workflows and the digitization of slides, among other advancements, providing an opportunity to implement diagnostic pipelines in research and ultimately aiming to reduce overall costs. Furthermore, proficiency in image analysis and R-scripts, along with expertise in histopathology and statistical analysis, is essential to fully realize the efficiency and potential of these digital tools.

Current patient consent forms enable numerous future projects with the same TMA blocks, producing at least 500 blank slides. The quantity is effectively doubled due to the availability of TMA clones, enabling hundreds of biomarker investigations.

Histomorphological tumor heterogeneity represents a challenging aspect of oncological research, affecting therapy and diagnosis [30]. While TMAs are debated for their accuracy in representing tumor heterogeneity, studies by Nocito et al., Camp et al., and Torhorst et al. demonstrate the representativeness of TMAs compared to whole-slides [23,24,31]. This study used three cores of 1.0 mm punch diameter per tumor for analysis, covering multiple times the tissue area than previously described methods in studies [23,24]. Core loss, due to inadequate tissue-paraffin adhesion, ranges from 4 to 25% [25,26,27,28,32,33]. The authors’ protocol of multiple melting cycles significantly reduced TMA core loss, though variability in institute-specific factors, such as tissue fixation methods, types of paraffin, FFPE block storage, antigen retrieval techniques, or the specific tissue entity employed in TMAs, limits comparison between studies to vague interpretations.

In terms of multimodal applications, TMAs are not only effective for IHC but are also compatible with other, more advanced techniques like in situ hybridization [34] and multiplex techniques [35]. The TMAs generated with this workflow are furthermore analyzed by Matrix-assisted Laser Desorption Mass Spectrometry Imaging (MALDI-MSI) [2,3,36], Cytometry by Time of Flight (CyTOF) [37,38], and Spatial Transcriptomics [39,40]. These methods validate research results and help bridge gaps between histomorphology, IHC, omics, and molecular pathology.

Regarding data analysis, QuPath, an open-source platform for tissue classification and staining evaluation in histopathological images using machine learning algorithms [29] was applied. The software proved effective for cell classification within individual TMA slides. However, its performance varied across different slides, necessitating the creation of separate classification models for each, an obstacle also highlighted in prior studies [29,41,42]. Despite these limitations, using QuPath for TMA analysis significantly reduced evaluation time. Analyzing a TMA with 30 patients took 52.5 min, totaling 15.75 h for all TMAs, compared to 70.9 h required for manual analysis, based on average onsite analysis duration. While verifying each cell’s classification in TMAs was time-consuming, it was an integral part of the quality control process. QuPath further supported the objective quantification of AB expression and structured data exportation, facilitating data management and streamlining subsequent statistical analysis.

The coded R-script was custom-fit for the data structure of QuPath’s results while remaining adaptable for future projects with other biomarkers or clinical data. The R-script auto-saves results as Kaplan–Meier survival curves and hazard ratios in an image format. Incorporating new projects into this framework requires minor adjustments, like updating file paths and accommodating clinical data differences. Proficiency in R and understanding the script’s architecture are necessary, limiting user-friendliness. Despite this, the script’s adaptability and speed in statistical analysis are advantageous. This is exemplified in the analysis of Cohort A, where the expression of six antibodies was investigated in both tumor and stromal cells across the entire cohort. The script’s adjustment to the project design and analysis took only two hours, resulting in high-quality figures and tables.

The CCCF-Biobank has already implemented the workflow to generate entity-specific TMA collections from samples within the CCCF Biobank and ISP. These TMAs are accessible to collaborating partners for research purposes. Partners benefit from the expedited workflow, by receiving publication-ready results in significantly less time compared to traditional methods.

## 5. Conclusions

In conclusion, the presented study outlines a scalable and reproducible workflow for efficient IHC biomarker investigations on large patient cohorts, from FFPE tissue blocks to publication-ready results. The workflow optimizes resource utilization and offers an alternative to traditional whole-slide IHC studies, resulting in quicker result generation, cost and time savings, and increased productivity and efficiency while maintaining result reliability. Despite requiring initial investment and expertise, this workflow can be adapted for broader use (i.e., metabolomics and spatial transcriptomics) in various high-throughput tissue-based studies, including biomarker discovery and validation. The method’s scalability positions it as a valuable tool for large-scale clinical research and diagnostic advancements, promoting more efficient data generation and enabling researchers to focus on data interpretation and application rather than extensive manual processing. The integration of AI in histopathological research workflows also paves the way for higher standardized protocols in pathology and related fields, ultimately contributing to more reliable and reproducible scientific outcomes.

## Figures and Tables

**Table 1:** Labeling the paraffin cassettes with DataMatrix barcodes improved their automatic detection by the microarrayer significantly (*p* < 0.01).

Test	Barcode-Detection				*t*-Test
	Without Label		With Labels		
	Detected Barcodes/ FFPE Blocks	Detection in %	Detected Barcodes/ FFPE Blocks	Detection in %	
1.	5/60	8.3%	54/60	90%	*p* < 0.001 *
2.	3/60	5.0%	50/60	83%	
3.	5/60	8.3%	52/60	86%	
4.	4/60	6.6%	53/60	88%	

* Two-sample *t*-test, two-tailed.

**Table 2:** Loss of cores on H&E slides: The TMAs of Cohort A underwent three melting cycles, resulting in an average of 4% of cores lost. Cohort B underwent six melting cycles, reducing the rate of core loss to 1.25%.

Cohort	Absolute Cores	Core Loss	Core Loss in %	Core Loss Within the Cohorts	
A TMA 1	170	6	3.5%	4%	*p* = 0.001 * * two-sample *t*-test, two-tailed
A TMA 2	139	5	3.5%		
A TMA 3	120	6	5.0%		
B TMA 1	165	4	2.4%	1.25%	
B TMA 2	168	3	1.7%		
B TMA 3	156	3	1.9%		
B TMA 4	132	1	0.7%		
B TMA 5	129	1	0.8%		
B TMA 6	123	0	0%		

**Table 3:** Schematic representation of the labor and material expenditure of studies using the presented workflow compared to the expenditure of similar whole-slide studies. The time estimates for whole-slide methods are based on an average of comparable whole-slide studies from the CCCF Biobank Freiburg over the period of three years.

Cohort A	TMAs	Whole-Slides
Number of blocks	2 TMAs	91 FFPE blocks
Number of IHC-stains	6	6
Slides required	12	546
Sectioning	40 min	2730 min (45.5 h)
IHC staining	480 min *	6552 min (109.2 h) *
Digitization	90 min	4095 min (68.25 h)
Necessary storage space	17.4 GB	791.7 GB
Evaluation of IHC	630 min (10.5 h)	2730 min (45.5 h)
Statistical analysis with R	120 min (2 h)	300 min (5 h)
Total working hours	22.67	273.45
Antibody amount	0.6 µg **	27.3 µg **
**Cohort B**	**TMAs**	**Whole-Slides**
Number of blocks	6 TMAs	305 FFPE Blocks
Number of IHC-stains	1	1
Slides required	6	305
Sectioning	20 min	1525 min (25.4 h)
IHC staining	240 min *	3660 min (61 h) *
Digitization	45 min	2287.5 min (38.1 h)
Necessary storage space	8.7 GB	442.25 GB
Evaluation of IHC	315 min (5.25 h)	1525 min (25.4 h)
Statistical analysis with R	60 min (1 h)	120 min (2 h)
Total working hours	11.25	164.6
Antibody amount	4.8 µg	244 µg

* manual staining including incubation times of 304 (Cohort A) and 152 min (Cohort B). ** Amount excluding LDH1A1 and VN due to lack of concentration information.

## Data Availability

Dataset available on request from the authors.
